# Leveraging Design and Management of Common Space of Senior Housing to Promote Social Connections Among Older Adults

**DOI:** 10.1093/geroni/igaf122.4365

**Published:** 2025-12-31

**Authors:** Astrid Jing Weinberg

**Affiliations:** Massachusetts Institute of Technology, Massachusetts Institute of Technology, Massachusetts, United States

## Abstract

The development of quality independent-living senior housing is a critical component for cities and towns to support residents to ‘age well’. This can contribute to a physical and social environment for older adults that reduces potential social isolation and loneliness and promotes healthy ageing. This study investigates the use of common areas within independent-living senior housing sites and its potential implications for residents’ social interactions and overall well-being. It draws upon a case study performed at three residential buildings in the ‘2Life Communities’ network in urban, suburban, and urban periphery settings within the Boston Metropolitan area, USA. Data was collected from residents (N = 153, CI = 95%) and staff members (N = 8), applying a random sampling strategy and a paper-and-pencil researcher-administered questionnaire for residents and the Delphi technique with a two-round online questionnaire for staff members. Additional site observations of the residents’ use of common spaces and related social interactions and behaviors were conducted. The study found similarities in the types of activities residents preferred to do when using common spaces as well as in their evaluations of community programs they most actively participated in. It also found that the design and availability of common spaces and activities varied across the three sites. Most participants stated satisfaction with their living environment and engagement in their community, though some noted infrequent use of common spaces or curated activities. It concludes with discussions on the value of common space and potential factors that could encourage active use of common areas by residents for enhancing social connectedness.

